# Phytocannabinoids—A Green Approach toward Non-Alcoholic Fatty Liver Disease Treatment

**DOI:** 10.3390/jcm10030393

**Published:** 2021-01-20

**Authors:** Klaudia Berk, Wiktor Bzdega, Karolina Konstantynowicz-Nowicka, Tomasz Charytoniuk, Hubert Zywno, Adrian Chabowski

**Affiliations:** Department of Physiology, Medical University of Bialystok, 15-089 Białystok, Poland; wbzdega@gmail.com (W.B.); karolina.konstantynowicz@umb.edu.pl (K.K.-N.); tomasz.charytoniuk@umb.edu.pl (T.C.); hubert.zywno@gmail.com (H.Z.); adrian@umb.edu.pl (A.C.)

**Keywords:** endocannabinoid system, endocannabinoid receptors, NAFLD, phytocannabinoids, marijuana, cannabidiol

## Abstract

Non-alcoholic fatty liver disease (NAFLD) is the most frequent chronic liver disease in adults in developed countries, with a global prevalence as high as one billion. The pathogenesis of NAFLD is a multifactorial and multi-step process. Nowadays, a growing body of research suggests the considerable role of the endocannabinoid system (ECS) as a complex cell-signaling system in NAFLD development. Although increased endocannabinoid tone in the liver highly contributes to NAFLD development, the complex effects and impacts of plant-derived cannabinoids in the aspect of NAFLD pathophysiology are yet not fully understood, and effective medications are still in demand. In our review, we present the latest reports describing the role of ECS in NAFLD, focusing primarily on two types of cannabinoid receptors. Moreover, we sum up the recent literature on the clinical use of natural cannabinoids in NAFLD treatment. This review is useful for understanding the importance of ECS in NAFLD development, and it also provides the basis for more extensive clinical phytocannabinoids testing in patients suffering from NAFLD.

## 1. Introduction

The term “non-alcoholic fatty liver disease” (NAFLD) involves simple fat accumulation in the liver and may progress to steatohepatitis, fibrosis, cirrhosis, and, in some cases, hepatocellular carcinoma (HCC) [1]. Over the past decade, the prevalence of NAFLD (nearly 30% in the general adult population) is increasing worldwide with each passing year due to sedentary lifestyles and the unlimited availability of fat- and calorie-rich diets in modern western society [2]. The incidence of NAFLD increases with age, with a tendency to occur in men before 50 years of age and women after 50 years of age [3]. NAFLD is considered the most frequent liver disease in the world, the second most common cause of liver transplantation, and a primary cause of the development of hepatocellular carcinoma. Given these facts and the lack of effective treatment, NAFLD is a relevant problem for all health systems. Unsurprisingly, the pathogenesis of NAFLD is associated with fat deposition in the liver. Particularly, increased accumulation of triacylglycerols (TAG) is characteristic of NAFLD development. Steatosis occurs as a result of the imbalance between lipid storage (from accelerated free fatty acids (FFA) influx and *de novo* synthesis) and hepatic lipid clearance (decreased oxidation of FFA in the liver and decreased synthesis of low-density lipoproteins (VLDLs)). The complex and multifactorial process of NAFLD development was explained initially by the “two hits” model. The “first hit” included hepatic steatosis as a consequence of metabolic syndrome and excessive TAG deposition in hepatocytes. The “second hit” seemed to be necessary to develop non-alcoholic steatohepatitis (NASH) from NAFLD. However, this model was too simple to fully describe the complexity of NAFLD. In 2010, Tilg and Moschen proposed the “multiple hit” model, suggesting that different risk factors such as insulin resistance, adipocytes dysfunction, nutritional factors, gut microbiota, and genetic and epigenetic factors act simultaneously on both intrahepatic and extrahepatic pathways, which finally leads to steatosis or inflammation [4]. The previous model assumed that NAFLD always precedes inflammation. According to the “multiple hit” model, depending on which signaling pathways are activated by risk factors, hepatic lipid overload or NASH development may occur [5]. Currently, there are only a few specific pharmaceutical strategies available to treat NAFLD. However, none of them is ideal. Many of the promising results from rodent studies on phytocannabinoids and the endocannabinoid system (ECS) have fueled hopes of implementing novel therapeutic approaches and targets in humans. In our review, we aim to discuss the latest reports describing the changes in the ECS and its components on the development and progression of NAFLD. Furthermore, we will summarize the clinical studies analyzing the effects of natural cannabinoids in NAFLD treatment.

## 2. Endocannabinoid System (ECS)

The endocannabinoid system is described as a widespread cellular signaling machine with an active physiological role in nearly all the organs and tissues. Initially, scientists distinguished two types of cannabinoid receptors (CBRs) and their endogenous ligands (anandamide, AEA, and 2-arachidonoyl glycerol, 2-AG) as well as two enzymes (fatty acid amide hydrolase (FAAH) and monoacylglycerol lipase (MAGL)) responsible for ligand metabolism. In the current literature, ECS has been expanded to an “-ome”; the concept of the endocannabinoidome (eCBome) includes not only endogenic ligands (eCBs) but also approximately 50 receptors and metabolic enzymes and more than 20 congeners of AEA and 2-AG with important physiopathological activities [6].

One of the major substances that may affect ECS activity are phytocannabinoids—chemicals found in *Cannabis* plants (marijuana). Despite the first reports of marijuana as an appetite inducer and pain killer derived from ancient civilizations, medical interest in marijuana usage in the context of ECS increased after isolation and characterization of the *Cannabis* major psychoactive component Δ9-tetrahydrocannabinol (THC) in 1964 [7]. After nearly 30 years, the main endogenously produced cannabinoids, AEA and 2-AG, were uncovered [7]. So far, their effects have been most widely studied in the central nervous system, where they modulate cognition, memory, learning, or energy balance [8,9]. Due to considerable advances in biotechnology, ECS components were detected in peripheral organs, including the liver.

In the neurons, eCBs produced “on-demand” are the retrograde synaptic messengers that, by preventing the development of excessive neuronal activity, play an important regulatory role. AEA and 2-AG are also present in the liver at concentrations comparable to the brain [10]; eCBs act as autocrine or paracrine agents through synthesis in a different or the same cell type, expressing cannabinoid receptor type 1 (CB1R). There is no doubt that AEA is synthesized from membrane phospholipid precursors, mainly by the sequential action of *N*-acyltransferase (NAT) and *N*-acyl-phosphatidylethanolamines-specific phospholipase D (NAPE-PLD), whereas the synthesis of 2-AG is regulated by two diacylglycerol lipases (DAGLα and β). The enzyme responsible for AEA degradation is FAAH, while 2-AG is hydrolyzed by MAGL. It is worth noting that eCBs also have biological features that extend beyond interaction with cannabinoid receptors. As an alternative pathway, eCBs may be oxidized by cyclooxygenase-2 (COX-2), distinct lipoxygenases (LOXs), or cytochromes P450 (CYPs) and therefore they may participate indirectly in inflammatory mediation [11,12]. Cannabinoids regulate biological processes by binding with different affinities to 7-transmembrane G protein-coupled-receptors, mainly cannabinoid receptors (CBRs) type 1 and type 2. The CB1 receptor was originally described as the “brain type” cannabinoid receptor because its levels of expression were high in the brain and central nervous system (CNS) [13]. However, in recent studies, lower levels of CB1R expressions were also found in peripheral nerves, the gastrointestinal tract, the cardiovascular system, and reproductive systems as well as in other tissues [14]. CB2 receptors are largely restricted to immune and hematopoietic cells, although functionally relevant expression has been found in specific regions of the brain and in the myocardium, the gastrointestinal tract, or reproductive cells [15]. CBRs constitute a family of transmembrane proteins that mediate many cellular processes. Stimulation of both CB1R and CB2R leads to activation of G_i/o_ protein-coupled receptors that, through inhibition of adenylyl cyclase activity, lead to a decrease in cAMP levels. They may also activate mitogen-activated kinases (MAPKs), including extracellular signal-regulated kinases 1/2 (ERK1/2), p38 mitogen-activated protein kinases, and c-Jun N-terminal kinase (JNK), as well as the phosphoinositide 3-kinase (PI3K)/protein kinase B (Akt) pathway. Depending on the circumstances, the outcome of CBR-mediated signaling could be the promotion of cell survival or cell death [16].

To date, we also know that several putative cannabinoid receptors mediate in the metabolic actions of cannabinoids. These include non-CB1R and non-CB2R cannabinoid-related orphan G protein-coupled receptors (GPCRs): GPR18, 55, and 119 [17,18,19].

Few investigations underlie the molecular mechanisms of GPCRs functions in the liver. GPR55 has been deeply explored in adipocytes. However, the direct signaling pathways of this receptor in hepatocytes have been unknown until recently. Studies on mice imply that GPR55 helps to sustain and/or reinforce insulin action in the liver cells [20]. Moreover, GPR55 might interact with CB2 in immune cells [21]. Considering this, researchers assumed that crosstalk between GPR55 and CB2 signaling may appear in tissues where both CB2 and GPR55 are co-expressed, including the liver, which may have an impact on hepatic glucose metabolism [22]. In the case of GPR119, it was presented that this receptor inhibited hepatic lipid accumulation by stimulating the phosphorylation of AMPK and consequently reduced the expression of transcription factors and enzymes involved in lipogenesis [23]. In contrast to GPR55 and 119, GPR18 is engaged in obesity-mediated inflammation, but there is a lack of evidence of its direct effects on hepatocytes [24].

Despite many inaccuracies in the understanding of the exact mechanisms of GPCR action, recent advances in research concerning pharmacological manipulation of these receptors assume that GPR18, 55, and 119 may be promising therapeutic targets for the prevention and treatment of metabolic disorders, including NAFLD [25,26].

CB1R and CB2R occur in high abundance in the central nervous system but have also been identified in human liver tissue. The major cannabinoid receptor present in hepatocytes, hepatic sinusoidal cells, or stellate cells is CB1R [27]. Currently, special attention has been paid to the CB1R isoform b (CB1Rb), which is absent in the brain and prominently expressed in hepatocytes and in pancreatic β-cells [28]. Generally, CB2R is widespread among cells of the immune system and is responsible for the immunomodulatory effects of cannabinoids and endocannabinoids [29]. Considering it in the liver, CB2R was identified mainly in Kupffer cells and stellate cells, and it is up regulated during pathology, especially occurring with inflammation such as NASH. However, the expression of this receptor in healthy hepatocytes is weak [30]. Although CBRs were believed to be functional only at plasma membranes, it is currently known that they are also expressed in intracellular organelle, e.g., mitochondria, where their role is not fully understood [31]. Experimental Wistar rats showed that expression of hepatic CBRs has a rhythmic daily pattern, with a higher expression of cannabinoid receptors during the light period [32]. What is more, plasma endocannabinoid concentrations also show a circadian rhythm, which could cause different CBR activation depending on the time of day [33].

Interestingly, the molecular basis of diurnal changes in cannabinoid receptors may be impacted by functional interactions with other receptor systems, such as orexin and other receptors [34]. For further consideration, it should be mentioned that both CBRs are species-dependent, which explains the discrepancy between their pharmacological effects in mice, rats, and humans. Therefore, their effects in one species cannot be directly extrapolated to others [35].

## 3. Cannabinoid System in Non-Alcoholic Fatty Liver Disease (NAFLD) Development and Progression—The Role of CB1R and CB2R and PPARs

By virtue of low CBR expression in the healthy liver, the endocannabinoid system in this tissue was considered to be non-meaningful. Nonetheless, various studies conducted on animal models and samples from the human liver revealed changes in CBR expression in many liver pathologies, prompting ECS to be recognized as a causative factor of this organ’s dysfunction [36].

The action of ECS, by inducing lipogenesis and inhibiting lipolysis in peripheral tissues as well as increasing appetite by anandamide and 2-arachidonoylglycerol, promotes fat accumulation that is the main feature of NAFLD occurrence [37,38,39]. Therefore, ECS overactivation accompanies metabolic pathologies such as obesity or hepatic steatosis. As shown in a rat model, the first changes in the endocannabinoid system that represent a higher risk of NAFLD development may already occur in prenatal life. Maternal high-fat diet feeding during the perinatal phase induced sex-specific long-term changes in the liver ECS components (i.e., increased protein expression of CB1R and CB2R) of the adult offspring [40]. The upregulation of hepatic CB1R in liver steatosis and fibrosis was repeatedly confirmed by human, animal, and in vitro studies [41,42]. What is more, the inhibition of CB1R eliminated these abnormalities, which is the ultimate proof for the significant participation of CB1R in the development of the above-mentioned disease [43]. CB1R affects lipid metabolism in the liver in many different ways. The activation of hepatic CB1R stimulated de novo lipogenesis through induction of the lipogenic transcription factor SREBP1c and its targets [41]. What is more, CB1R is also linked with stearoyl-CoA desaturase-1 (SCD1) activity. According to Liu et al., high fat diet induced (HFD) hepatic steatosis and insulin resistance development occurred bidirectionally. HFD upregulated SCD1 activity and, as a result, increased the production of monounsaturated fatty acids (MUFA), which inhibited FAAH activity and increased hepatic AEA levels. Moreover, increased endogenous levels of MUFA activated CB1R, which promoted de novo lipogenesis through induction of lipogenic gene expression including SCD1, creating a positive feedback loop [44]. Additionally, CB1R also regulates fatty acid oxidation. CB1 −/− mice had higher basal CPT1 mRNA levels than wild-type mice, and treatment with CB1R antagonist decreased the CPT1 activity [45]. Interestingly, the inhibition of CB1R diminished hepatic fatty acid uptake by the regulation of FAT/CD36 expression [46]. Otherwise, recent studies conducted on mice demonstrated a novel molecular mechanism of CB1R action—downregulation of perlipin 2 (PLIN2) by CB1R knockout leads to suppression of lipogenesis and TAG synthesis [47]. These findings prompt further research exploring pharmacologic modulation of the CB1-PLIN2 axis, which might represent a novel therapeutic approach for NAFLD treatment.

The role of CB1R has also been described in insulin resistance (IR), which is one of the most common metabolic abnormalities associated with NAFLD. Considering the epidemiological data, nearly 70–80% of individuals with obesity or type 2 diabetes mellitus (T2DM) suffer from simple steatosis [1,48]. As one of the “multiple hits”, IR plays a crucial role in the establishment of lipotoxicity, oxidative stress, and inflammation development in the liver [49]. Consequently, in patients suffering from NAFLD, genetic, extracellular, and intracellular factors disturb insulin signaling pathways, contributing to maintenance and worsening the inappropriate response of cells to insulin [50]. It still remains an open question of whether insulin resistance is the cause or consequence of hepatocytes lipid overload [49].

As shown by O’Hara et al., CB1R activation in the central nervous system is sufficient for the induction of insulin resistance in the liver [51]. However, several studies showed that treatment with non-brain penetrant CB1R antagonists favoring the peripheral CB1R action improved glycemic control, body weight, and metabolic profile in obese patients. Undoubtedly, CB1R is involved in the modulation of basal insulin secretion and glucose-stimulated insulin secretion from β pancreatic cells [52]. Furthermore, CB1R-mediated increase in glycogenolysis and/or gluconeogenesis resulted in the increased hepatic glucose production that is revealed in primary hepatocytes [53]. Moreover, CB1R overexpression interrupted insulin signaling and clearance, leading to insulin resistance development in mouse livers [54]. Because changes in CB1R expression interfere with the insulin transmission pathway, they may also indirectly contribute to liver steatosis. Scientists suspect that, in obesity, CB2R may also magnify fatty liver progression by inducing adipose tissue inflammation and insulin resistance development in peripheral tissues [55]. Studies conducted on diet-induced obesity (DIO) mice showed that knocking out the *Cnr2* gene encoding CB2R declined hepatic steatosis and improved peripheral insulin sensitivity [38].

Studies on CB2R focus on its immune-modulatory character, but this receptor may also be involved in diet-induced metabolic changes [56]. However, the exact CB2R role in NAFLD and metabolic disorder development is unclear because literature data in this context are contradictory [55]. The changes in CB2R-dependent pathways significantly contribute to the development of insulin resistance and NAFLD but with a different mechanism of action than the CB1R [57]. Because of the peripheral localization (immune system, bones, lungs, gastrointestinal tract, testicles) of CB2R, its action in the central nervous system is less pronounced than CB1R [58,59]. As shown in in vitro experiment on HepG2 cells, CB2R directly modulated lipid metabolism by targeting genes involved in lipid synthesis and by elevating the expression of CB1R [42]. What is more, the genetic inactivation of CB2R reduced the steatosis and liver triacylglycerol concentration caused by high-fat diet in mice [57]. Interestingly, tissue fractionation in this study revealed that, during obesity development, the elevated expression of the *Cnr2* gene was derived mainly from the non-parenchymal liver cells, while the expression of this gene in hepatocytes was inappreciable [57]. However, research on human liver biopsies uncover the CB2R expression on hepatocytes in NAFLD patients but not in the healthy liver [60]. Therefore, whether *Cnr2* gene expression or CB2R expression is present in healthy hepatocytes remains an open question. There is no doubt, however, that the expression of CB2R varies in a state of impaired lipid metabolism in humans. Currently, it is known that CB2R mRNA level correlates positively with key hepatic lipogenic enzyme gene expression from fatty acid de novo synthesis pathway, namely Acetyl-coenzyme A carboxylase 1 (ACC1) in the liver-derived from an obese woman [61]. These data showed that activation of CB2R caused lipotoxicity and took part in NAFLD progression. However, this research did not answer the question of whether CB2R modulated lipid uptake and fatty acid transport. Interestingly, increased CB2R mRNA is linked with the elevation of hepatic pro-inflammatory molecules (IL6, TNFα, resistin) as well as anti-inflammatory adiponectin, suggesting a dual role of CB2R in liver pathologies [61].

In addition to reversible hepatic steatosis, CBRs are also involved in the further mechanism of NAFLD deterioration such as innate immune activation, inflammation, cell death, or fibrosis leading to progressive liver damage [19]. Most studies used CBRs agonists/antagonists to interrogate the important role of these receptor isoforms in liver failure [62]. In the case of CB1R, it was reported that antagonism of this receptor diminished hematopoietic stem cell (HSC) activation and proinflammatory cytokine production in mice with HFD-induced NAFLD [63,64]. Additionally, in a rodent study, it was noted that inhibition of CB1R reduced hepatic fibrosis through decreased TGF-ß production or collagen deposition in cirrhotic livers [64]. However, by CB2R occurrence in immune cells, its role in the inflammation was more widely discussed. Antifibrogenic CB2R properties in the human liver were first presented by Boris Julien et al. [65]. Studies conducted by Guillot et al. on cultured Th17 lymphocytes revealed that activation of CB2R reduced profibrogenic cytokine, namely interleukin 17 production (IL-17), but not antifibrogenic interleukin 22. Moreover, in macrophages and hepatic myofibroblasts, which also express CB2R, activation of this receptor reduced the proinflammatory response. Knowing that progression of NAFLD is connected with various events, the antifibrogenic properties of CB2R result from not only antiproliferative and antiapoptotic effects on myofibroblasts and inhibited Kupffer cell proinflammatory response but also on inhibition of IL-17 release that induces immune and fibrogenic effects in the liver [66]. In line with this conclusion are studies where chronic CB2R stimulation with selective agonist contributed to the regression of liver fibrosis. Observed changes were associated with diminished liver inflammation and collagen deposition, and consequently reduced fibrosis [67].

In recent years, it has been suggested that non-cannabinoid receptors such as peroxisome proliferator-activated receptors (PPARs) are also part of the endocannabinoid system [68]. These nuclear factors have three isotypes (PPARα, PPARß/δ, PPARγ), and they are characterized by different tissue distribution patterns and ligand specificities, which highlight their distinct functions [69]. In general, PPARs are crucial in the genetic regulation of the complex pathways of mammalian metabolism, including lipid and glucose homeostasis, inflammation, and cell proliferation [70]. Preclinical as well as clinical studies have demonstrated that PPARs play a key role in regulating adipogenesis and inhibiting liver fibrosis and, through them, NAFLD development [19].

Since 2002, evidence has existed that endocannabinoids, endocannabinoid-like compounds, phytocannabinoids, and synthetic cannabinoid ligands bind to and activate PPARs [68]. The mechanisms of PPAR activation by individual phytocannabinoids have been summarized by O’Sullivan et al., who revealed that THC and CBD activate PPAR γ and do not activate PPARα [69]. Importantly, there are still many cannabinoids whose activity regarding PPARs is not clear. What is more, it is still unknown why some cannabinoids exert the same physiological effects, one through the PPAR action and the other through a different receptor, although both have the same ability to activate PPARs. It appears that there are still many unestablished factors that could influence the interactions between cannabinoids and PPARs [69].

## 4. Phytocannabinoids

The *Cannabis sativa* plant is rich in a broad spectrum of phytochemicals including cannabinoids, terpenoids, sterols, and flavonoids. Phytocannabinoids (natural cannabinoids contained in the *Cannabis* plant) are considered to be the most active ingredients of marijuana that may be found in the human body of all the above mentioned. Most of the phytocannabinoids are found in female *Cannabis* inflorescence [70].

Among many phytocannabinoids found in *Cannabis*, the most studied agents are Δ9-tetrahydrocannabinol (THC), cannabidiol (CBD), and tetrahydrocannabivarin (THCV) [71].

There are plenty of *Cannabis sativa* varieties (cultivars) existing, and each one has an individual combination of bioactive compounds. In this case, especially important is the proportion of THC:CBD:THCV, which is the cause of the unique and different pharmacodynamic and medicinal properties of various *Cannabis* extracts [72]. *Cannabis* also contains a large number of acidic precursors of the aforementioned molecules, respectively: Δ9-tetrahydrocannabinolic acid (THCA), cannabidiolic acid (CBDA), and tetrahydrocannabivarinic acid (THCVA). These compounds may reveal interesting therapeutic properties, such as attenuation of body weight gain and amelioration of glucose-insulin homeostasis in a mouse model of HFD-induced obesity after administration of THCA [73]. However, the current knowledge in the field of phytocannabinoid acids is limited and requires further examination [74]. Therefore, the effect of medicinal *Cannabis* should be considered as the “entourage effect” of *cannabis* as a whole [70]. When analyzing the effects of the individual phytocannabinoids presented in our review, their complex pharmacology should be considered. The different effects on the response of several phytocannabinoids studied *in vivo* are possibly related to the competition and displacement of endogenous cannabinoids, with the different centers (orthosteric and allosteric) and with the biased signalling of cannabinoid target receptors [72]. Additionally, phytocannabinoids interact with each other. For instance, CBD has the ability to antagonize THC effects by CBR1 and non-CB1 receptor mechanisms of action. However, CBD may also potentiate some THC effects in an additive or synergistic fashion [75].

Promising research results regarding clinical application of cannabinoids in many morbidities and increasing acceptance of the clinical use of marijuana and its derivatives has led the pharmaceutical industry to research new compounds based on *Cannabis* [76]. This was accompanied by a growing awareness of the role of the endocannabinoid system in our body. Currently, THC and CBD are the two major active compounds of Cannabis. Table 1 provides a summary of the main opposing characteristics of these substances that account for the differential effects exerted on the human body.

## 5. Effects of Prolonged *Cannabis* Use in the Context of NAFLD and its Comorbidities

Progressive legalization of marijuana across the world allowed researchers to observe its interesting properties in encouraging or counteracting many metabolic and psychiatric diseases. Although *Cannabis* use is generally considered an unhealthy, addictive habit, there is growing strong evidence that it can be protective against the development of metabolic disturbances leading to hepatic steatosis and its progression.

This is a particularly interesting fact because dysregulation of the endocannabinoid system is undoubtedly one of the most important factors in the development of NAFLD. Moreover, *Cannabis* use is expected to increase over the coming years as a new therapeutic agent for many disorders.

**Figure 1:**
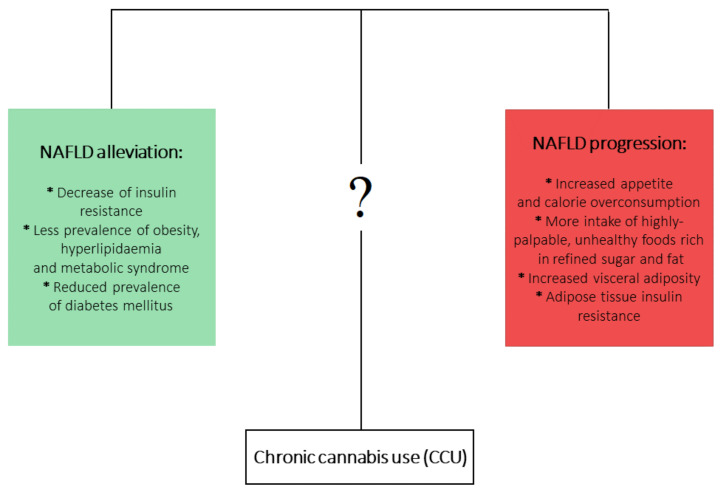
The opposite metabolic effects of chronic *Cannabis* use (CCU).

On the other hand, it is well documented that chronic *cannabis* use (CCU) has been associated with metabolic disturbances ascertained as detrimental factors leading to NAFLD development. Firstly, CCU is undoubtedly linked with increased appetite and calorie overconsumption [86,87]. What is more, this appetite dysregulation is augmented by a higher intake of highly-palpable, unhealthy foods that are rich in refined sugar and fat [88,89,90]. On the other hand, a multitude of studies analyzing the metabolic effects of CCU observed plenty of beneficial effects that could counteract the development of NAFLD. It has been shown that prolonged *Cannabis* use is linked with decreased prevalence of insulin resistance [91,92] and hyperlipidemia and metabolic syndrome [93,94], as well as decreased frequency of diabetes mellitus occurrence [89]. In the aspect of CCU and obesity, an overwhelming majority of studies have shown a decreased prevalence of obesity among marijuana users. However, one study showed increased visceral adiposity in *Cannabis* smokers, but this study considered only 30 *cannabis* smokers [95,96,97]. Mounting evidence indicates that current or past marijuana use is associated with a lower risk of NAFLD development, regardless of the presence of metabolic risk factors [95,98,99,100]. At first glance, this association is contradictory to the role of ECS in NAFLD development. Although it is clearly proven that increased endocannabinoid tone in liver and brain relates to obesity and metabolic syndrome and contributes to the development of NAFLD, the complex effects and impact of phytocannabinoids in relation to NAFLD pathophysiology are not yet fully clear (Figure 1).

One of the possible mechanisms responsible for the positive influence of *Cannabis* use on the prevalence of NAFLD and other metabolic diseases may include the antagonistic action of CBD and THCV on CB1R [101,102]. Additionally, CBD has been described as acting as a negative allosteric modulator of CB1R in HEK 293A and in STHdhQ7/Q7 cells, two model systems that highly express CB1R [90]. Antagonism of CB1R improves the insulin sensitivity of hepatocytes [45], decreases intrahepatic triglyceride synthesis [41], and decreases secretion of very-low-density lipoprotein (VLDL) [27]. Diminished IR may contribute to improved hepatic steatosis, hepatomegaly, and metabolic syndrome by restoring the optimal hepatic glucose metabolism and decreasing liver fat accumulation [49].

Moreover, the anti-inflammatory effects of phytocannabinoids should be emphasized, as they can inhibit secretion of pro-inflammatory cytokines (TNF-a, IL-6) and adipokines (leptin) and lead to the upregulation of inflammatory mediators such as cell transcription factor (NF-kB) [63]. Excessive inflammatory response plays a robust role in NAFLD development and progression to NASH [103]. CBD has been shown to alleviate liver inflammation induced by a high fat-cholesterol (HFC) diet in mice by inhibition of NF-kB and can likewise restrain NLRP3 inflammasome activation, which both lead to a reduction in inflammatory response [104,105,106]. Another possible mechanism that is involved in the beneficial properties of *Cannabis* is development of tolerance and down-regulation of CB1R from repetitive THC use. THC should theoretically induce or worsen NAFLD by its agonistic role regarding CB1R. However, it has been proven that repetitive use of THC may result in decreased CB1R density, which may contribute to a dose-dependent inverse relationship between marijuana use and NAFLD occurrence [78,93,107]. Finally, the most important factor is that *Cannabis* is a source of not only THC, CBD, and THCV but also various other phytocannabinoids such as cannabidivarin (CBDV), cannabigerol (CBG), cannabigerovarian (CBGV), cannabigerolic acid (CBGA), and cannabinol (CBN). The therapeutic potential of these compounds remains largely unexplored. Thus, there is a need for further research directed at establishing whether phytocannabinoids are indeed ‘a neglected pharmacological treasure trove’ [78,108]. Perhaps, without knowledge about the exact amount and the strain of *cannabis* used by study participants, it will not be possible to clearly assess which of the phytocannabinoids contained in marijuana are responsible for the beneficial or harmful effects related to the pathogenesis of NAFLD (Table 2). Therefore, more research is needed to evaluate which of the phytocannabinoid and non-cannabinoid receptors are responsible for those positive effects related to prolonged marijuana use and NAFLD occurrence [98,109].

## 6. THC and NAFLD

Δ9-tetrahydrocannabinol (THC) is the main active compound of *Cannabis sativa* and is responsible for its psychoactive effect. THC acts as a partial agonist at CB1R and CB2R and as an agonist of the transient receptor potential channel of the vanilloid 2 subtype (TRPV-2) [79]. THC is useful in the treatment of many diseases associated with a lack of appetite such as chemotherapy-induced nausea, AIDS, and anorexia nervosa due to its antivomitic and orexigenic effect. It is also used in the treatment of non-cachexic diseases such as chronic pain, neuropathic pain, spasticity due to multiple sclerosis, and other neurological disorders [80,86,111,112].

In some of the medical conditions listed above, the orexigenic properties of THC are extremely useful. Conversely, in individuals suffering from obesity and metabolic syndrome, the action of THC will be highly undesirable because, acting as a CB1R agonist in the central nervous system, THC increases the qualitative ratings of hunger, leading to calorie overconsumption [113,114]. THC exacerbates endocannabinoid tone in the liver not only by direct agonism at CB1R/CB2R, but also by increasing total AEA and 2-AG levels in hepatocytes by competing with them in binding to fatty acid binding protein-1 (FABP-1), making eCBs less available for enzymatic degradation and hydrolysis [115]. In the light of the cited studies, it is highly probable that THC promotes development of NAFLD, although there is a lack of studies focusing strictly on THC. Considering the impact of marijuana on the liver and the pro-steatotic effects of THC as a constituent of *Cannabis*, it may be suspected that protective properties are exerted by factors other than the THC phytocannabinoids present in *Cannabis*. Given the cumulative impact of marijuana, the alleged adverse, pro-steatotic effects of THC as a constituent of *Cannabis* are apparently offset by other phytocannabinoids [116].

## 7. CBD and NAFLD

The second abundant component of Cannabis extracts, CBD, functions as a non-competitive negative allosteric modulator of CB1R and an inverse antagonist of CB2R [77,101]. CBD is also an inverse agonist for the orphan G-protein coupled receptors 3, 6, 12, 18, and 55 (GPR3, GPR6, GPR12, GPR 18, and GPR55) [79,117].

Despite crossing the blood-brain barrier (BBB), CBD does not cause psychoactive effects (distinct like CB1R agonists such as THC); consequently, it lacks abuse potential. Additionally, though its agonistic influence on CB1R, CBD does not cause depressive effects characteristic of rimonabant, a withdrawn antiobesity drug, acting as an inverse antagonist of CB1R [75]. CBD, due to its easy availability on the market (there are plenty of unverified formulations sold as dietary supplements), is widely advertised as a potentially supportive factor in the treatment of various diseases, although its properties in suggested indications are often questionable, except for the control of refractory seizures [118]. Cannabidiol generally exerts no serious side effects, but if any of these appear, the ones most often reported are gastrointestinal problems or somnolence [119,120]. It seems that the only particular concern about acute high dosing of CBD is the risk of hepatotoxicity and drug interactions. These effects were clearly demonstrated in a mouse model where CBD induced hepatotoxicity in doses that were scaled for mouse equivalent doses from the maximum recommended human CBD dose in EPIDIOLEX^®^ (20 mg/kg) [121]. In fact, the clinical characteristic of EPIDIOLEX^®^ (a drug containing an oral solution of CBD) confirms mild risk of hepatic enzymes elevation, especially in patients with coexisting moderate to severe hepatic impairment and/or were being treated in combination with hepatotoxic drugs [84]. However, in in vivo studies on murine models of NAFLD, CBD havs shown an anti-oxidative, anti-inflammatory, and hepatoprotective effect [122,123,124]. Studies conducted by Silvestri et al. have shown its substantial antisteatotic effectiveness, as CBD was able to directly dose and time dependently reduce a ccumulated intracellular lipid levels [125]. In the cited study, a human hepatosteatotic model was established using liver Human Hepatocyte Line 5 (HHL-5) cells, where steatosis was induced by incubation with oleic acid. What is more, the observed effects were independent of CB1 or TRPV1 receptor activation but were probably caused by post-translational modification of various proteins: extracellular signal-regulated kinases 1/2 (ERK1/2), the cAMP-response element binding protein (CREB), the proline-rich Akt substrate of 40 kDa (PRAS40), AMP-activated protein kinase alpha2 (AMPKa2), and the signal transducer and activator of transcription proteins (STATs), which are the key factors controlling hepatic lipid metabolism [125].

Th antisteatotic effect of CBD in the liver has also been confirmed by an in vivo model of ob/ob mice, where CBD administration at a dose of 3 mg/kg for 4 weeks substantially reduced liver TAG content [125].

High anti-inflammatory efficiency of CBD was confirmed in high-fat/high-cholesterol (HFC) fed mice, where CBD alleviated liver inflammation through decreased inflammation pathway protein expression, namely NF-κB, p65, and NLRP3 inflammasome [105]. Another study confirmed that CBD can reduce the extent of liver inflammation, oxidative/nitrative stress, and cell death triggered by the ischemia/reperfusion mechanism. CBD suppressed the acute inflammatory response by the inhibition of Kupffer cells and liver sinusoidal endothelial cell activation (ICAM-1), which led to decreased pro-inflammatory cytokines (TNF-α) and chemokine (CCL3, CXCL2) expression, and likewise diminished the delayed inflammatory cell infiltration [126]. To sum up (Table 3), numerous studies pronounced that CBD treatment may give dual benefits in dealing with NAFLD, not only by decreasing hepatosteatosis but also by relieving associated inflammation, which would protect from NAFLD progression and complications in which excessive inflammation plays a crucial role [127]. Additionally, it has been proposed that targeting NF-κB and NLRP3 inflammasome pathways in macrophages, with the use of CBD, might be a novel treatment method for the fibrotic complication, NASH [110].

Despite the very promising results of in vitro and in vivo animal studies, clinical studies have not confirmed any spectacular properties of CBD in relation to NAFLD treatment.

Important data about the possible positive influence of CBD comes from the completed phase II of a clinical study that evaluated the effects of GWP42003 (which is an acronym of CBD, a title given by GW Pharma—the company responsible for the study) on liver fat accumulation and other metabolic parameters in patients with fatty liver disease, compared to a placebo group. Participants self-administered CBD orally, in a daily dose of 200/400/800 mg (depending on the group) for 8 weeks. At the end of the investigation, liver triacylglycerol levels measured by MRI scan did not differ significantly. Moreover, mean total serum cholesterol concentration and total serum triacylglycerol were not significantly decreased, and there was no change in the ratio of HDL-C to LDL-C. During the trial, serious adverse effects were not disclosed, and the main reported mild adverse effects were diarrhea, dyspepsia, and nausea [128].

Another pilot clinical study determined the efficacy and safety of CBD alone, or in combination with THCV in patients with type 2 diabetes. Patients were divided into five groups that administered, respectively, 100 mg of CBD, 100 mg of CBD combined with 5 mg of THCV, 5 mg of CBD and 5 mg THCV, only 5 mg THCV, or only placebo. After 13 weeks of treatment, in the groups treated with CBD alone or in combination with THCV, the liver triacylglycerol accumulation measured by MRI scan did not differ significantly. There was also no significant difference in mean total serum triglyceride or cholesterol concentration. However, in a group of subjects treated with 100 mg of CBD or 5 mg CBD + 5 mg THCV, mild deterioration occurred, as participants significantly gained in subcutaneous and internal fat [129] (Table 4). These cases demonstrate that studies on cell/animal models do not always predict direct clinical effects and pharmacology in humans, which underlies the need for clinical testing compounds in vivo before stating a conclusion on their suitability for a given purpose [75].

The possible reason for the lack of a decent therapeutic effect in clinical studies may be the low dose of CBD. The doses used in the cited clinical studies ranged from 100 to 800 mg/day, which, in the case of a 70 kg individual, will equal 1.4 to 11.4 mg/kg/day. Moreover, plasma concentrations were not measured in any study, thus it is not clear how much administered CBD reached the bloodstream. Rodent studies typically used 2.5 to 10 mg/kg/day, and most of the metabolic positive effects were seen at higher doses [130,131]. However, the question should be asked whether increasing the dose of CBD to one that caused a metabolic effect in mice will not cause unacceptable side effects, which would affect inpatient’s low compliance.

Finally, it is worth noting that the mechanism of CBD activity observed in some of the cited studies may be not associated with the direct effects on CB receptors, but through inverse agonism for non-CB1R and non-CB2R cannabinoid-related orphan G protein-coupled receptors (GPCRs), GPR3 and GPR12, or by agonism of GPR55. [117,132] All of these receptors are expressed in the liver and despite the fact that their exact function in hepatic lipid metabolism is currently not fully known, it is worth mentioning. For instance, GPR3 or GPR12 knock-out mice displayed increased adiposity and liver fat accumulation [117]. Studies on GPR55 have an ambiguous character. In an experiment conducted by Lipina et al., GPR55 knock-out mice developed obesity, and it displayed a reduction in insulin signaling capacity [20]. On the other hand, in some research, no tendency to increased adiposity was seen in GPR55 knock-out mice compared to wild type mice [133,134]. Development of agents affecting these orphan receptors may be an approach that could provide new therapeutic possibilities [117].

## 8. THCV and NAFLD

Tetrahydrocannabivarin (THCV), after CBD, is known as a non-psychoactive analog of THC presented in *Cannabis*. It has interesting pharmacodynamic properties in humans, as it can act on cannabinoid receptors in a dose-dependent manner. In low doses, it acts as a neutral antagonist of CB1R and a partial agonist of CB2R, while in high doses it acts as a CB1R/CB2R agonist. Moreover, THCV is also acting on GPR55 and transient receptor potential channels (TRP), but the effects of THCV on this type of receptors are relatively less well documented [75,80,130]. In the presence of other cannabinoids, THCV acts as a competitive antagonist of CB1R. It can reverse many of the effects mediated by THC in vivo, as it was shown to antagonize CB1R and CB2R mediated effects in mice models in the presence of THC or anandamide [78,102]. Furthermore, THCV was shown to diminish the THC-mediated tachycardia and psychogenic effects in humans, which confirms its antagonistic potential on CB1R. At the same time, it does not cause the depressive effects associated with central CB1R antagonism [135].

There are in vivo studies (Table 5) that have shown that THCV can be a promising agent in preventing the development of NAFLD and its complications. In research using obese mice, THCV showed many positive properties in the context of counteracting NAFLD. Therapy with THCV improved glucose-insulin metabolism, reduced body weight, decreased appetite, and, most importantly, reduced hepatic lipid accumulation [136]. Supporting this data are studies conducted on human hepatocytes, where THCV was effective in reducing intracellular triacylglycerol levels [125]. The molecular mechanism of THCV action that contributes to its therapeutic effect remains unknown. However, in vitro studies proposed that beneficial effects of THCV were due to antagonism of CB1R, mainly in the liver and adipose tissue, while other reports claimed that THCV acted directly on hepatocytes, without impact on any known receptor [125]. In human hepatocytes, THCV lowered intracellular lipid levels and stimulated lipolysis through activation of AMPK2a, STATs, and ERK1/2, which are the key transcription factors regulating the cellular energy metabolism [125]. The above mentioned positive properties of THCV have led to the commencement of several clinical studies in which the effect of this compound on improving metabolic parameters in diabetic patients was examined [129]. Some improvements in glycemic parameters have been observed in the group administering 5 mg of THCV through 13 weeks versus placebo—a decrease in fasting glucose levels and amelioration in insulin β cell function measured by HOMA2 index [129]. However, none of the cited clinical studies (Table 6) showed a significant improvement in liver lipid accumulation. The lack of ameliorating effects in the liver triglyceride accumulation may be a consequence of low THCV dose (5 mg) [129]. Perhaps the application of a higher dose could bring some significant effects in lipid metabolism improvement, bearing in mind the fact that doses that effectively reduced liver steatosis in rats were significantly higher (12.5 mg/kg) [136]. It is worth emphasizing that the use of higher doses of THCV in humans did not cause any notable side effects [135]. What is more, a study with healthy humans demonstrated that oral administration of 10 mg THCV can positively modulate brain neuronal regions that are associated with appetite dysregulation and obesity development. Thus, it could be suspected that THCV may be an anti-obesity medication [137]. Undoubtedly, more research is needed (perhaps using higher doses) to determine whether THCV can find a place in NAFLD therapy.

## 9. Conclusions

Our review comprehensively summarized investigations showing the involvement of two CBR types in NAFLD development and progression. It seems that these components of the ECS system may be a valuable target in the treatment of NAFLD and its systemic complications. Furthermore, based on the presented clinical studies, we demonstrated that the pharmacological effect of phytocannabinoids counteracting NAFLD exist. However, the lack of therapeutic methods in hepatosteatosis treatment motivates scientists to investigate the possibilities of ECS action modifications, the system that is strongly engaged in NAFLD pathophysiology. Extending knowledge about the usefulness of natural cannabinoids in NAFLD and its comorbidities treatment seems extremely important, bearing in mind that obesity and its metabolic sequelae are a 21st-century global epidemic.

## Figures and Tables

**Table 1:** Comparison of selected features of tetrahydrocannabinol (THC) versus cannabidiol (CBD).

Feature	THC	CBD	Reference
Interaction with receptors: a) CB1R b) CB2R	+ (partial agonist) - (weak antagonist)	- (negative allosteric modulator) - (inverse agonist)	[77,78,79]
Psychoactive effect	Yes	No	[80]
Appetite stimulation	Yes	No	[80]
Cardiovascular stimulation (inducing tachycardia and hypertension)	Yes	No	[80]
Anticonvulsant effect	Yes	No	[80]
Therapeutic indications approved by the FDA ^1^	Anorexia associated with weight loss in AIDS ^3^ patients Nausea and vomiting associated with anticancer chemotherapy Multiple sclerosis spasticity	Lennox-Gastaut syndrome Dravet syndrome	[81,82,83,84]
Formulations available on US pharmaceutical market	Nabilone (trade name Cesamet) synthetic THC analog aviable as oral capsule Dronabinol (trade name Marinol)—synthetic formulation of the main THC constituent enantiomer found in *Cannabis*: [(−)-trans-Δ9-tetrahydrocannabinol] as an oily resin in capsules	Epidiolex^®^—pharmaceutical formulation of CBD as an oral solution	[82,83,84]
Combination drugs available on US ^2^ pharmaceutical market	Nabiximol (trade name Sativex)—oral spray standardized in composition, formulation, and dose, delivering of 2.7 mg THC and 2.5 mg CBD per dose.		[85]

^1^ FDA, Food and Drug Administration; ^2^ US, United States; ^3^ AIDS, Acquired Immunodeficiency Syndrome.

**Table 2:** Clinical studies analyzing the effects of *Cannabis* use on Non-alcoholic fatty liver disease (NAFLD) progression and development.

Main Outcome	Research Model	Participants	Method	Reference
↓ prevalence of NAFLD	Population-based case-control study	5,950,391 *Cannabis* users from the HCUP-NIS ^7^ database.	Presence of NAFLD diagnosis among patients.	[110]
↓ prevalence of NAFLD	Cross-sectional study	22,366 adult participants of US NHANES ^8^ and NHANES III surveys. Healthy adults (>20 years) with no history of excessive alcohol abuse, viral hepatitis or pregnancy. Age, gender, and ethnicity-adjusted analysis. Current and past users of *Cannabis* compared to non-users, independent of metabolic risk factors	Hepatic steatosis examination: Serum alanine aminotransferase (ALT) Liver ultrasonography	[97]
No difference in intrahepatic fat content and OGTT ^3^. ↑ % of abdominal visceral fat	Cross-sectional, case-control study	60 patients of JH-BPRU ^9^ in Baltimore and CRC-NIH ^10^ in Bethesda *Cannabis* smokers (*n* = 30) and control subjects (*n* = 30) matched for age, sex, ethnicity, and BMI ^11^.	Abdominal and intrahepatic fat content were assesed by MRI and MRS ^4^ scan. Insulin-sensitivity was evaluated with OGTT.	[82]
↓ prevalence of metabolic syndrome	Cross-sectional study	8478 adult participants of NHANES survey. Current and past *Cannabis* users	Prevalence of metabolic syndrome examined by biochemical markers, BP ^14^ and waist circumference	[81]
↓ fasting insulin levels ↓ prevalence of IR ^1^ ↓ waist circumference	Cross-sectional study	4657 participants of NHANES survey. Current and past *Cannabis* users	Prevalence of insulin resistance by HOMA-IR ^5^ calculation. Assesment of waist circumference.	[78]
↓ prevalence of obesity	Cross-sectional study	52,375 participants of NESARC ^12^ and NCS-R ^13^ surveys of US nationally representative samples. Current and past *Cannabis* users	Prevalence of obesity examined by BMI calculation.	[80]
↓ prevalence of NAFLD	Prospective cohort study	838 HIV-HCV ^15^ coinfected patients, adjusted for BMI, hazardous alcohol consumption and current or lifetime use of lamivudine/zidovudine. Current and past *Cannabis* users	NAFLD examination using liver ultrasonography	[99]
↓ prevalence of IR	Follow-up study	703 HIV-HCV coinfected patients Current and past *Cannabis* users	Prevalence of insulin resistance by HOMA-IR calculation.	[79]
↓ prevalence of NAFLD↓ overtime increment of steatosis score No difference in fibrosis score	Follow-up study	390 patients with diagnosed non-affective psychosis. Current and past *Cannabis* users	NAFLD and NASH examination using clinical FLI ^6^ score.	[86]
↓ prevalence of T2DM ^2^ in *Cannabis* users	Cross-sectional study	10,896 adult participants of NHANES III survey. Current and past *Cannabis* users	Prevalence of T2DM defined based on self-report or abnormal glycaemic parameters	[76]
No increase in prevalence or progression of hepatic fibrosis	Meta-analysis	5,973,595 participants of 9 studies including patients with NAFLD/HCV/HCV-HIV coinfection Current and past *Cannabis* users	Prevalence and progression of hepatic fibrosis among *Cannabis* users	[100]

^1^ IR, Insulin resistance; ^2^ T2DM, Diabetes mellitus type 2; ^3^ OGTT, Oral glucose tolerance test; ^4^ MRS, Magnetic resonance spectroscopy; ^5^ HOMA-IR, Homeostatic model assessment for insulin resistance; ^6^ FLI, Fatty liver index; ^7^ HCUP-NIS, Healthcare Cost and Utilization Project-National Inpatients Sample database; ^8^ NHANES, The National Health and Nutrition Examination Survey; ^9^ JH-BPRU, Johns Hopkins Behavioral Pharmacology Research Unit in Baltimore; ^10^ CRC-NIH in Bethesda-Clinical Research Center, The National Institute of Health Center in Bethesda; ^11^ BMI, Body Mass Index; ^12^ NESARC, National Epidemiologic Survey on Alcohol and Related Conditions; ^13^ NCS-R, National Comorbidity Survey Replication; ^14^ BP, Blood Pressure; ^15^ HIV/HCV, Human Immunodeficiency Virus/Hepatitis C Virus; ↓, decrease; ↑, increase.

**Table 3:** Studies analyzing the in vitro and in vivo effects of CBD in NAFLD and related diseases.

Main Outcome	Research Model	Dose of CBD	Reference
Reduced TAG levels in HHL5 ^1^ cells, independently of CB1R or TRPV1R action Reduced TAG content of larvae yolk sacs Reduced liver TAG ^8^ content in ob/ob mice	HHL5 cell culture Zebrafish ^2^ embryos and larvae Female ob/ob mice	10 μM of CBD for 3 days 5 μM of CBD for 3 days 3 mg/kg for 4 weeks	[125]
CBD alleviated lipid accumulation and steatohepatitis induced by HFC ^5^ diet (↓ of serum ALT and TAG concentration, ↓ of hepatic TAG content in group treated with HFC+CBD versus HFC-only)	Male C57BL/6J mice fed with HFC diet and normal diet as control	5 mg/kg of CBD for 8 weeks	[92]
CBD attenuated markers of I/R-induced hepatic injury: ALT ^6^, AST ^7^, proinflammatory chemokine, cytokine, and adhesion molecule expression, NF-κB ^4^ activation, cell necrosis and inflammatory cell infiltration.	Male C57BL/6J mice CB2 −/− mice exposed for hepatic I/R ^3^	3 and 10 mg/kg of CBD	[123]
Dose-dependent decrease in rats weight gain	Male Wistar rats	2.5 and 5 mg/kg/day for 14 days	[124]

^1^ HHL5, Human hepatocyte line 5; ^2^ Zebrafish, Danio rerio; ^3^ I/R, ischemia/reperfusion; ^4^ NF-κB, nuclear factor kappa-light-chain-enhancer of activated B cells; ^5^ HFC diet, high-fat cholesterol diet; ^6^ ALT, alanine transaminase; ^7^ AST, aspartate transaminase, ^8^ TAG, triacylglycerols; ↓, decrease.

**Table 4:** Clinical studies analyzing the effects of CBD on liver fat accumulation.

Main Outcome	Research Model	Participants and CBD Dose	Method	Reference
No significant changes in visceral adiposity or liver TAG assessed by MRI/MRS after treatment.	Randomized, double-blind, placebo-controlled, parallel group pilot study	62 subjects with T2DM treated with 200 mg CBD daily or placebo for 13 weeks	MRI/MRS ^1^ scan to examine the liver fat content at the beginning and at end of treatment visit	[126]
Mean liver TAG levels did not significantly (*p* > 0.05) differ between the CBD and placebo groups.	Randomized, partially blind, placebo-controlled, dose-ranging phase 2 clinical study	25 participants with NAFLD treated with 200/400/800 mg of CBD daily or placebo for 8 weeks	MRI/MRS ^1^ scan to examine the liver fat content at the baseline and at end of treatment visit	[125]
Mean % of liver fat did not change significantly (*p* > 0.05) after treatment.	Randomized, double blind, placebo controlled, parallel group, phase 2 clinical study	13 participants with T2DM treated with 100 mg CBD daily or placebo for 13 weeks	MRI/MRS ^1^ scan to examine the liver fat content at the baseline and at end of treatment	[105]

^1^ MRI/MRS, Magnetic Resonance Imaging/Magnetic Resonance Spectroscopy.

**Table 5:** In vitro and rat in vivo studies analyzing the effects of THCV in NAFLD and its comorbidities development.

Main Outcome	Research Model	Dose of THCV	Reference
Reduction of body mass, an increase of energy expenditure, improvement of glucose tolerance, and amelioration of insulin resistance. No significant effect on liver TAG content. Increase of energy expenditure, significant reduction of liver TAG content.	-DIO ^1^ mice -ob/ob mice	12.5 mg/kg of THCV for 30 days 12.5 mg/kg of THCV for 30 days	[129]
Reduced TAG levels in HHL5 cells, independently of CB1R or TRPV1R action. Reduced TAG content of larvae yolk sacs	-HHL5 ^2^ cell culture -Zebrafish ^3^ embryos and larvae	10 μM of THCV for 3 days 10 μM for 3 days	[125]
Decrease in body weight, hypophagia	Male C57 BL6 mice	3 mg/kg of THCV for 2 days	[131]

^1^ DIO, Diet-induced obesity; ^2^ HHL5, Human hepatocyte line 5; ^3^ Zebrafish, Danio rerio.

**Table 6:** Clinical studies analyzing the effects of THCV in NAFLD and related disorders development.

Main Outcome	Research Model	Participants	Method	Reference
Mean % of liver fat did not change significantly (*p* > 0.05) after treatment	Randomized, double blind, placebo controlled, parallel group, phase 2 clinical study	13 participants with T2DM treated with 5 mg of THCV daily or placebo for 13 weeks	MRI/MRS ^1^ scan to examine the liver fat content at the baseline and at end-of-treatment	[105]
No significant changes in liver TAG concentration or visceral adiposity in any of the treatment groups.	Randomized, double-blind, placebo-controlled, parallel group pilot study	62 subjects with T2DM treated with 5 mg of THCV daily or placebo for 13 weeks	MRI/MRS scan to examine the liver fat content at the beginning and at end-of-treatment visit	[126]
Increase of neural responding to rewarding and aversive stimuli	Double-blind clinical study	20 healthy participants received 10 mg of THCV daily or placebo	fMRI ^2^ scan to examine neural response to rewarding and aversive food stimuli	[130]

^1^ MRI/MRS, Magnetic Resonance Imaging/Magnetic Resonance Spectroscopy; ^2^ fMRI, Functional Magnetic Resonance Imaging.
